# Fluorination Site and Degree Regulate the Decomposition of Fluorinated Ethyl Acetate Solvents on Lithium Metal: A First-Principles Molecular Dynamics Study

**DOI:** 10.3390/nano16130810

**Published:** 2026-06-30

**Authors:** Fuming Du, Shuting Hu, Xiao Wang, Xin Gu, Jianjun Liu, Hailong Hu

**Affiliations:** 1 School of Materials Science and Engineering, Hunan Institute of Technology, Hengyang 421002, China; 2 State Key Laboratory of High Performance Ceramics and Ultrastructure, Shanghai Institute of Ceramics, Chinese Academy of Sciences, Shanghai 200050, China; 3 Research Institute of Aerospace Technology, Central South University, Changsha 410083, China

**Keywords:** lithium metal batteries, fluorinated ethyl acetate, solid electrolyte interphase, density functional theory, ab initio molecular dynamics

## Abstract

Fluorinated carboxylate ester solvents are promising electrolyte components for lithium metal batteries because they can improve oxidative stability and promote LiF-rich solid electrolyte interphase (SEI) formation. However, how fluorination position and degree regulate their intrinsic decomposition behavior on lithium metal remains unclear. Herein, density functional theory (DFT) calculations and ab initio molecular dynamics (AIMD) simulations were employed to systematically investigate six pure fluorinated ethyl acetate solvents on the Li(001) surface, including α-fluorinated ethyl fluoroacetate (EFA), ethyl difluoroacetate (EDFA), and ethyl trifluoroacetate (ETFA), as well as β-fluorinated 2-fluoroethyl acetate (FEA), 2,2-difluoroethyl acetate (DFEA), and 2,2,2-trifluoroethyl acetate (TFEA). Electronic-structure analysis shows that although the lowest unoccupied molecular orbitals (LUMOs) of all six solvents are mainly distributed around the carbonyl and adjacent regions, the dominant electron-accepting center strongly depends on the fluorination position. In α-fluorinated solvents, the LUMO is highly localized on the α-C atom directly bonded to fluorine, whereas in β-fluorinated solvents, it remains concentrated around the carbonyl C atom. Real-time Bader charge and bond-evolution analyses reveal that fluorination position is the primary factor governing the initial decomposition pathway. The α-fluorinated series preferentially undergoes C-F bond cleavage, and increasing fluorination degree induces deeper cascade decomposition; fully fluorinated ETFA even exhibits C=O double bond cleavage. In contrast, β-fluorinated solvents preferentially undergo carbonyl-side C-O bond cleavage, while C-F bond cleavage occurs only in subsequent steps or is completely suppressed. Notably, β-fluorinated solvents retain high chemical stability even with α-H atoms because the LUMO electron density on α-H is negligible. Meanwhile, limited deep decomposition can still provide F^−^ species for SEI formation. These findings establish an atomic-level structure–reactivity relationship for fluorinated carboxylate ester solvents and provide theoretical guidance for designing stable electrolyte solvents for lithium metal batteries.

## 1. Introduction

Lithium metal batteries (LMBs) are promising next-generation energy-storage systems because metallic lithium offers an ultrahigh theoretical capacity (3860 mAh/g) and the lowest redox potential (−3.04 V vs. the standard hydrogen electrode) [1,2,3]. However, the high reactivity of Li metal toward organic electrolytes causes continuous parasitic reactions, low Coulombic efficiency, dendrite growth, and safety risks. Therefore, constructing a compact and mechanically robust solid electrolyte interphase (SEI) is essential [4,5,6,7]. Linear carboxylate esters, including ethyl acetate (EA), methyl propionate (MP), and fluorinated ester derivatives, are attractive electrolyte solvents because of their low viscosity, low melting point, and structural tunability, which are particularly beneficial for low-temperature batteries [8,9,10,11,12,13]. Nevertheless, conventional carboxylate esters often show poor compatibility with Li metal because acidic α-H atoms adjacent to the carbonyl group can trigger condensation, polymerization, and uncontrolled organic-rich SEI formation [14,15,16]. Fluorination is therefore a useful molecular-engineering strategy: F substituents can regulate frontier molecular orbitals, solvation behavior, reductive pathways, and LiF-rich interphase formation [17,18,19].

Previous studies have shown that fluorinated carboxylate ester-based electrolytes can improve low-temperature operation, high-voltage stability, solvation structure, and interphase chemistry in lithium batteries [20,21,22,23]. More recently, molecular engineering of fluorinated EA derivatives demonstrated that both fluorination site and degree can regulate solvent stability, physicochemical properties, and battery performance [24]. In particular, β-fluorinated EA derivatives can remain chemically stable toward Li metal despite retaining α-H atoms, suggesting that fluorination position may be more decisive than simply increasing the number of F atoms. This observation motivates a direct atomic-level comparison between α- and β-fluorinated ethyl acetate solvents.

Although these experimental advances provide valuable guidance for electrolyte design, most studies have been conducted in multicomponent electrolyte systems containing lithium salts, co-solvents, and film-forming additives. Such formulations are closer to practical battery electrolytes, but the coexistence of multiple reactive components makes it difficult to isolate the intrinsic decomposition behavior of the fluorinated ester solvent itself. In particular, the atomic-level differences between α- and β-fluorinated ethyl acetate solvents during their initial reduction on Li metal surfaces remain insufficiently understood. Several key questions remain unresolved: whether C-F bond cleavage or C-O bond cleavage occurs first, how the fluorination position changes the preferred electron-injection region, and why fully fluorinated α- and β-isomers exhibit markedly different decomposition tendencies. Clarifying these issues is essential for establishing a reliable structure–reactivity relationship for fluorinated carboxylate ester solvents.

Computational methods, especially density functional theory (DFT) and ab initio molecular dynamics (AIMD), provide powerful tools for probing electrolyte/electrode interfacial reactions at the atomic scale [25]. These methods can reveal molecular orbital characteristics, charge-transfer processes, bond-breaking events, transient intermediates, and initial SEI precursor formation that are difficult to directly capture experimentally [26,27,28,29]. For example, DFT and AIMD simulations were also employed to clarify the decomposition mechanisms of ether- and fluorinated ether-based electrolytes on Li metal surfaces in our previous studies, revealing the important roles of molecular fluorination, interfacial electron transfer, and bond-cleavage pathways in regulating early-stage SEI chemistry [30,31].

**Figure 1:**
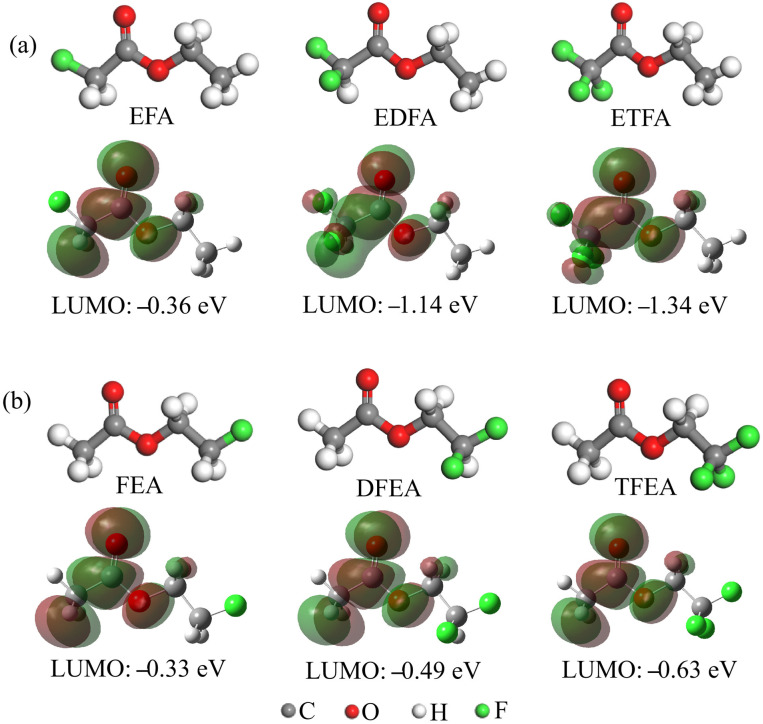
Molecular structures, lowest unoccupied molecular orbital (LUMO) distributions, and energy levels of six fluorinated ethyl acetate solvents. (**a**) EFA, EDFA, and ETFA. (**b**) FEA, DFEA, and TFEA. For each molecule, the ball-and-stick model is shown above, and the LUMO distribution plot with an isosurface value of 0.02 a.u. is shown below, with the corresponding LUMO energy level (in eV) labeled. Gray, red, white, and green spheres represent carbon, oxygen, hydrogen, and fluorine atoms, respectively.

Herein, we present a systematic DFT and AIMD study on the intrinsic decomposition mechanisms of six fluorinated ethyl acetate solvents on the Li(001) surface. Three α-fluorinated solvents, namely EFA, EDFA, and ETFA, and three β-fluorinated solvents, namely FEA, DFEA, and TFEA, are comparatively investigated. The molecular structures and LUMO distributions of the six solvents are shown in Figure 1. To exclude the interference of lithium salts, co-solvents, and additives, pure solvent/Li interface models are employed. By analyzing frontier molecular orbitals, interfacial charge transfer, decomposition product distributions, and atomistic bond-cleavage pathways, this work aims to clarify how fluorination site and fluorination degree regulate the electronic structure and reductive reactivity of fluorinated ethyl acetate solvents. Particular attention is paid to the initial decomposition pathways of α- and β-fluorinated derivatives and the formation of inorganic and organic SEI precursors. The results provide molecular-level insights into the structure-dependent decomposition behavior of fluorinated carboxylate ester solvents on Li metal surfaces and offer theoretical guidance for the rational design of advanced electrolyte solvents for high-performance lithium metal batteries.

## 2. Computational Methods

### 2.1. Geometry Optimization and Frontier Orbital Analysis of Isolated Solvent Molecules

The molecular geometries of all fluorinated ethyl acetate solvents were optimized using Gaussian 09 [32]. Geometry optimizations were performed at the B3LYP/6-311+G(d,p) level of theory [33]. Frequency calculations were subsequently carried out at the same theoretical level to confirm that all optimized structures corresponded to true local minima, as indicated by the absence of imaginary vibrational frequencies. The lowest unoccupied molecular orbital (LUMO) distributions of the optimized solvent molecules were visualized using GaussView 5. The isosurface value for orbital visualization was set to 0.02 a.u. These isolated-molecule calculations were used to compare the intrinsic electron-accepting characteristics of the α-fluorinated series and the β-fluorinated series, before constructing the corresponding Li-metal interface models.

### 2.2. Ab Initio Molecular Dynamics Simulations of Pure-Solvent/Li-Metal Interfaces

#### 2.2.1. Construction of Interface Models

Interface models were constructed following our previously reported Li-metal interface strategy [30,31], with the full technical protocol provided in Section S1 of the Supplementary Materials. Briefly, a seven-layer 4 × 4 Li(001) slab was used as the metallic substrate, with the bottom two Li layers fixed to mimic bulk Li, and a fixed He buffer layer plus a vacuum region was introduced along the surface-normal direction to reduce artificial periodic interactions.

For a consistent comparison of intrinsic solvent reactivity, each interface contained 17 solvent molecules with the same initial solvent skeleton arrangement, average density, and solvent–surface configuration. Only the fluorination position and degree were varied for different interface models. The initial EDFA layer was generated by amorphous-cell sampling and force-field pre-optimization, and the EFA, ETFA, FEA, DFEA, and TFEA models were obtained by substituting the corresponding F/H atoms. The corresponding interface configurations are presented together with the AIMD simulation results in Section 3.1.1 and Section 3.2.1. After atomic substitution, all interface models were further geometry-optimized before the AIMD simulations, as described below.

#### 2.2.2. DFT/AIMD Parameters and Post-Processing Analysis

All interface geometry optimizations and AIMD simulations were performed using VASP 5.4.4. The projector augmented wave (PAW) method was used to describe the ion–electron interactions, and the exchange–correlation energy was treated using the Perdew–Burke–Ernzerhof generalized-gradient approximation (PBE-GGA) functional [34,35,36,37]. During interfacial calculations, the bottom two Li layers and the helium buffer layer were fixed.

For geometry optimizations, a plane-wave kinetic-energy cutoff of 520 eV and a Γ-point 1 × 1 × 1 k-point mesh were used. The structures were optimized using the conjugate-gradient algorithm until the residual force on each relaxed atom was less than 0.05 eV/Å. Bader charge analysis was performed based on static single-point calculations using a 2 × 2 × 1 Monkhorst–Pack k-point mesh. AIMD simulations were carried out at the Γ point with a reduced cutoff energy of 400 eV. The electronic self-consistent-field convergence criterion was set to 10^−4^ eV. AIMD simulations were performed in the NVT ensemble at 330 K using a Nosé–Hoover thermostat. A time step of 1 fs and a total simulation time of 20 ps were used for all systems. No lithium salt, co-solvent, or additive was included, allowing the intrinsic decomposition behavior of the six fluorinated ethyl acetate solvents on Li(001) to be directly compared.

Bond-length evolution during AIMD simulations was analyzed using in-house Python scripts (Python 3.10) to identify bond-cleavage events and decomposition pathways. Bader charge analysis was performed using the Henkelman-group Bader code (version 1.05), based on the method developed by Henkelman and co-workers [38,39], to quantify interfacial charge transfer and evaluate the electronic evolution during solvent decomposition.

## 3. Results and Discussion

### 3.1. Intrinsic Reactivity of α-Fluorinated Ethyl Acetate Solvents on the Lithium Metal Surface

#### 3.1.1. Decomposition Products and Charge Transfer of the α-Fluorinated Interfaces

To evaluate the intrinsic reductive stability of α-fluorinated ethyl acetate solvents on Li metal, three pure solvent/Li interface models containing 17 EFA, EDFA, or ETFA molecules were constructed with identical initial spatial distributions.

**Figure 2:**
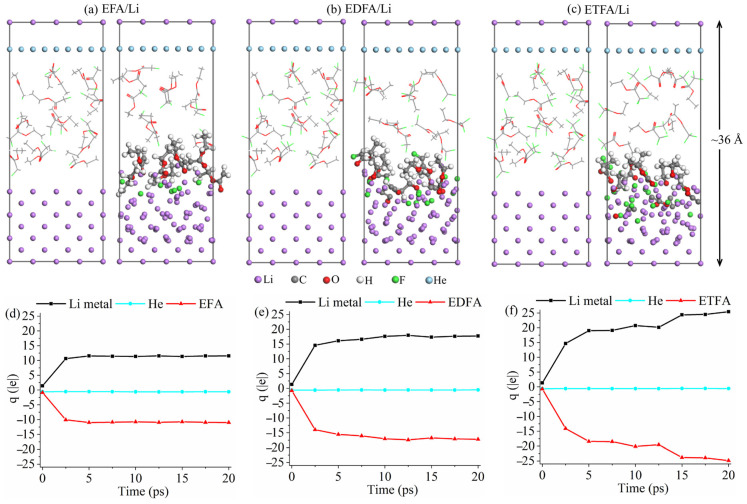
Initial configurations, final configurations after 20 ps AIMD simulation, and real-time charge transfer of α-fluorinated ethyl acetate/lithium metal interfaces. (**a**) EFA/Li system; (**b**) EDFA/Li system; (**c**) ETFA/Li system. (**d**–**f**) Real-time charge evolution curves of lithium metal, helium layer, and solvent molecules in the corresponding interface systems, respectively. In panels (**d**–**f**), black square, cyan circle, and red triangle curves represent Li metal, He layer, and solvent molecules, respectively. Undecomposed solvent molecules in the simulation are represented by line models, while decomposed solvent fragments, lithium metal layers, and helium atom layers are represented by ball-and-stick models.

As shown in Figure 2a–c, after 20 ps of AIMD simulation, seven solvent molecules decomposed in each system, but the decomposition depth and product distribution varied significantly with α-fluorination degree. The number of F^−^ ions increased from seven in EFA to eleven in EDFA and sixteen in ETFA, while ETFA additionally generated two O^2−^ species. The number and diversity of organic fragments also increased from EFA to ETFA. EFA mainly produced CH_2_COOCH_2_CH_3_ fragments, whereas EDFA generated CHCO, OCH_2_CH_3_, CHCOOCH_2_CH_3_, and related intermediates. In ETFA, smaller and more deeply reduced fragments, such as CC, CCO, and OCH_2_CH_3_, were observed. These results indicate that increasing α-fluorination promotes not only C-F bond cleavage but also subsequent C-O and C=O bond cleavage, leading to deeper decomposition on the Li surface.

Bader charge analysis was further performed to monitor interfacial charge transfer (Figure 2d–f). The He buffer layer remained nearly neutral throughout the simulations, confirming the reliability of the charge analysis. All three systems showed rapid electron transfer from the Li slab to the solvent layer within the first 5 ps, followed by gradual equilibration. The EFA/Li interface reached a nearly stable charge state after ~5 ps, EDFA/Li approached equilibrium within ~10 ps, whereas ETFA/Li required more than 15 ps, indicating more prolonged electron uptake and more extensive decomposition.

The equilibrium charge transfer increases monotonically with α-fluorination degree, with the net positive charge on the Li slab increasing from approximately +12 |e| for EFA/Li to +18 |e| for EDFA/Li and +24 |e| for ETFA/Li. This trend is consistent with the progressive lowering of the LUMO energy level in the α-fluorinated series (Figure 1a), which facilitates electron injection from the Li surface into the solvent molecules. Together with the increased formation of F^−^ and O^2−^ species, these results indicate that higher α-fluorination enhances the intrinsic reductive reactivity of ethyl acetate solvents on Li metal. The representative decomposition pathways of EFA, EDFA, and ETFA are discussed below.

#### 3.1.2. Decomposition Trends of the α-Series

**Figure 3:**
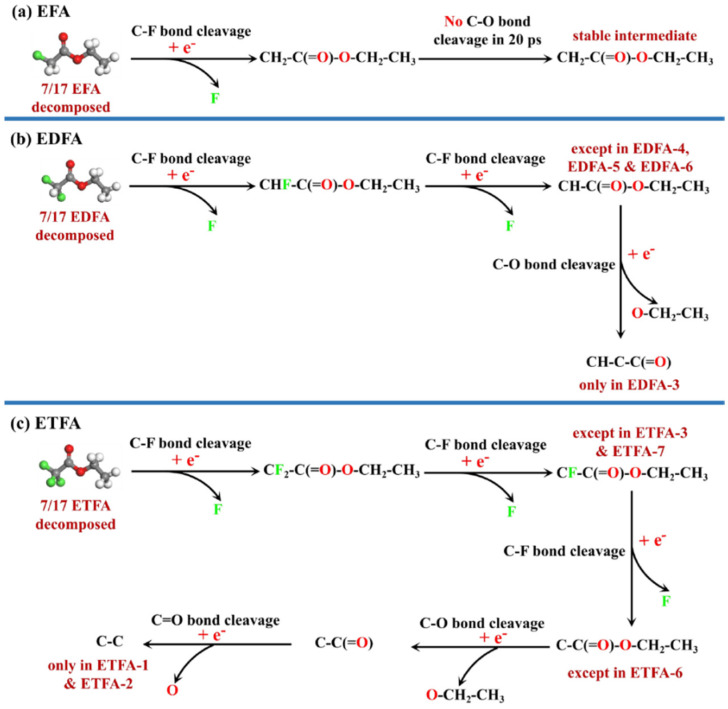
Schematic summary of the representative decomposition trends of α-fluorinated ethyl acetate solvents on the Li(001) surface. Black arrows indicate bond-cleavage sequences; red text highlights electron-involved steps, oxygen-containing fragments, and trajectory-specific notes; green text highlights fluorine-containing fragments.

The bond-length evolutions and representative decomposition snapshots for EFA, EDFA, and ETFA are provided in the Supplementary Materials (Figures S1–S6). Here, the discussion focuses on the comparative decomposition trend across the α-fluorinated series, as summarized in Figure 3 and Table 1. Overall, the α-series is characterized by preferential C-F bond cleavage, and the decomposition depth increases progressively with increasing α-fluorination degree.

EFA represents the mildest α-fluorinated case. As shown in Figure S1, all seven decomposed EFA molecules underwent only single C-F bond cleavage within the 20 ps AIMD trajectory, producing F^−^ and CH_2_COOCH_2_CH_3_-type defluorinated organic fragments. These fragments remained intact during the subsequent simulation and did not undergo further carbonyl-side C-O cleavage, C=O cleavage, or α-C-H cleavage within the present time window. Therefore, CH_2_COOCH_2_CH_3_ can be regarded as a stable defluorinated intermediate on the AIMD timescale, indicating that direct defluorination is the dominant initial pathway when only one α-F atom is present.

Increasing the α-fluorination degree to EDFA promotes successive C-F bond cleavage and, in the most reactive trajectory, further carbonyl-side C-O bond rupture. The representative EDFA-3 trajectory shows stepwise cleavage of the two C-F bonds followed by C2-O1 bond cleavage, yielding F^−^ together with CHCO- and OCH_2_CH_3_-type fragments (Figure S4). This sequence suggests that complete defluorination can make the ester framework more susceptible to subsequent electron-induced fragmentation.

ETFA exhibits the deepest decomposition among the α-fluorinated solvents. In the most reactive ETFA trajectories, rapid cleavage of three C-F bonds is followed by C-O bond cleavage and, in ETFA-1 and ETFA-2, further C=O double-bond cleavage (Figure S6). The generation of both F^−^ and O^2−^ indicates that fully α-fluorinated ETFA can efficiently provide LiF- and Li_2_O-related inorganic precursors. However, this enhanced inorganic precursor formation is accompanied by severe solvent fragmentation. Overall, the α-series follows a fluorination-degree-dependent cascade decomposition trend: C-F cleavage occurs first, while higher α-F substitution progressively enables deeper C-O and C=O bond activation.

### 3.2. Intrinsic Reactivity of β-Fluorinated Ethyl Acetate Solvents on the Lithium Metal Surface

#### 3.2.1. Decomposition Products and Charge Transfer of the β-Fluorinated Interfaces

**Figure 4:**
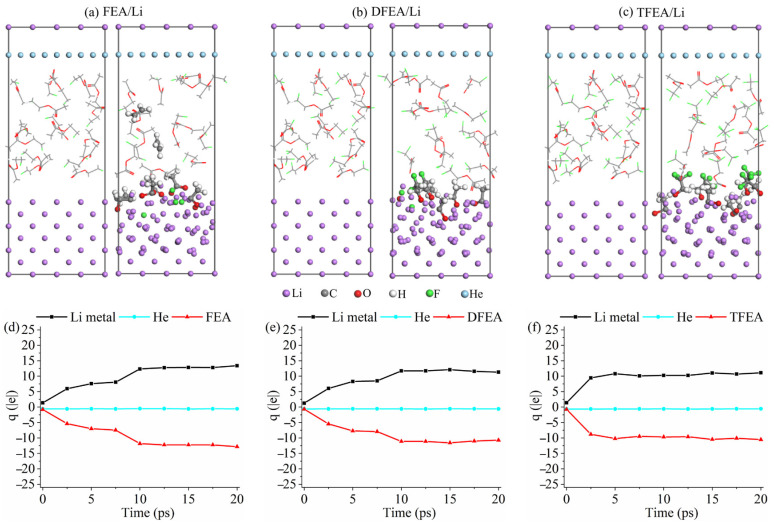
Initial configurations, final configurations after 20 ps AIMD simulations, and real-time charge-transfer profiles of β-fluorinated ethyl acetate/Li metal interfaces. (**a**) FEA/Li system; (**b**) DFEA/Li system; (**c**) TFEA/Li system. (**d**–**f**) Time-dependent charge evolution of the Li metal slab, He buffer layer, and solvent molecular layer in the corresponding interface systems. In panels (**d**–**f**), black square, cyan circle, and red triangle curves represent Li metal, He layer, and solvent molecules, respectively. Undecomposed solvent molecules are shown as line models, whereas decomposed solvent fragments, Li metal atoms, and He atoms are shown as ball-and-stick models.

To clarify the effect of fluorination position on interfacial reactivity, 20 ps AIMD simulations were performed for three β-fluorinated ethyl acetate solvents, namely FEA, DFEA, and TFEA, on the Li(001) surface. Compared with the α-fluorinated systems, the β-fluorinated solvents showed much lower decomposition probabilities, with only four, three, and four molecules decomposed in the FEA, DFEA, and TFEA systems, respectively (Figure 4a–c). Moreover, unlike the α-series, where decomposition became more severe with increasing fluorination degree, the β-series did not exhibit a monotonic increase in interfacial reactivity.

Product distributions and charge-transfer profiles further highlight the distinction between α- and β-fluorination. FEA and DFEA generate limited F^−^/O^2−^ species only after secondary reactions, whereas TFEA mainly produces CH_3_CO and OCH_2_CF_3_-type fragments without detectable C-F cleavage. The equilibrium Li-slab charge (Figure 4d–f) is approximately +13 |e| for FEA/Li and only approximately +11 |e| for DFEA/Li and TFEA/Li, which are much lower than those of EDFA/Li and ETFA/Li. These results indicate that β-fluorination does not lower the effective reduction center as strongly as α-fluorination. Instead, the LUMO remains dominated by the carbonyl region, so electron injection preferentially activates the carbonyl-side C-O bond rather than the β-C-F bonds (Figure 1b).

#### 3.2.2. Decomposition Trends of the β-Series

**Figure 5:**
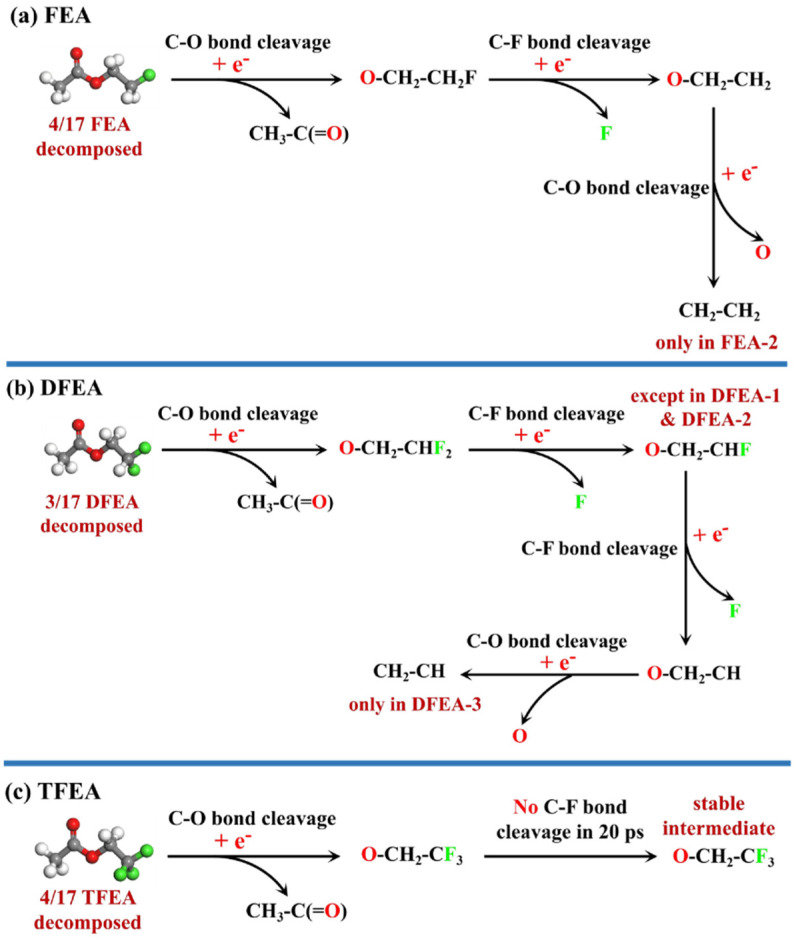
Schematic summary of the representative decomposition trends of β-fluorinated ethyl acetate solvents on the Li(001) surface. Black arrows indicate bond-cleavage sequences; red text highlights electron-involved steps, oxygen-containing fragments, and trajectory-specific notes; green text highlights fluorine-containing fragments.

Detailed bond-length evolutions and representative decomposition snapshots of the β-fluorinated solvents are shown in the Supplementary Materials (Figures S7–S13). Compared with the α-fluorinated series, the β-series exhibits a distinct decomposition feature: the initial decomposition is mainly triggered by carbonyl-side C-O bond cleavage, whereas β-C-F bond cleavage is delayed, adsorption-configuration-dependent, or completely suppressed. The representative decomposition pathways and product/intermediate distributions of FEA, DFEA, and TFEA are summarized in Figure 5 and Table 1.

For FEA, the representative pathway shown in Figure 5a is initiated by cleavage of the carbonyl-side C-O bond, followed by secondary C-F bond cleavage in the reduced fluorinated ethoxy fragment. In this trajectory, this initial cleavage produces CH_3_CO- and OCH_2_CH_2_F-type fragments. Subsequent reduction of the fluorinated ethoxy fragment can generate F^−^ together with an OCH_2_CH_2_-type fragment. In the most extensively decomposed FEA-2 trajectory, additional cleavage of the ethoxy-side C-O bond was also observed (Figure S9). Notably, an alternative sequence initiated by C-F bond cleavage can occur when the β-F-containing ethoxy group is directly exposed to the Li surface (Figure S10), indicating an adsorption-configuration-dependent pathway, as detailed in the Supplementary Materials. These results explain why FEA exhibits relatively large charge transfer within the β-series, while still differing fundamentally from α-fluorinated EFA, whose decomposition is directly initiated by α-C-F bond cleavage.

DFEA shows slower and less frequent decomposition than FEA. The decomposed DFEA molecules are initiated by carbonyl-side C-O bond cleavage rather than direct defluorination. Secondary C-F cleavage occurs only in the more extensively reduced alkoxy fragments (Figure S12), indicating that defluorination is not the primary decomposition step for DFEA. TFEA is even more resistant to defluorination: it undergoes only single carbonyl-side C-O bond cleavage to form CH_3_CO- and OCH_2_CF_3_-type fragments, while all three β-C-F bonds remain intact during the 20 ps AIMD trajectory. Therefore, the β-series demonstrates that increasing the fluorination degree does not necessarily enhance reductive decomposition when the F atoms are spatially separated from the dominant electron-accepting carbonyl/C-O region.

The contrasting reactivity can be rationalized by considering the LUMO energy and localization together with the stabilization of the resulting fragments. In the α-series, progressive fluorination lowers the LUMO energy and localizes the electron-accepting region on the α-C atom and adjacent carbonyl framework, allowing interfacial electrons to directly populate orbitals coupled to the α-C-F bonds. The resulting F^−^ species are readily stabilized by nearby Li^+^ ions, while continued electron uptake after defluorination can further activate the carbonyl-side C-O bond and, in ETFA, even the C=O bond. In contrast, the fluorinated carbon in the β-series is spatially separated from the carbonyl-centered LUMO, leaving little electron-accepting density on the β-C-F bonds and causing electron injection to preferentially weaken the carbonyl-side C-O bond. This spatial decoupling explains both the absence of a monotonic increase in charge transfer and decomposition with increasing β-fluorination and the lack of α-C-H cleavage, because the LUMO population around the retained α-H atoms is negligible and C-H cleavage would generate less favorable hydride-like products than the LiF- and Li-containing alkoxide/oxide-like species formed through C-F and C-O cleavage. Thus, α-H-related reactions are not dominant under the present early-stage Li/solvent interfacial conditions, consistent with the improved Li-metal compatibility reported experimentally for β-fluorinated EA-based electrolytes [24].

### 3.3. Implications for Early SEI Precursors and Practical Electrolyte Environments

The different fragment distributions observed in the six solvent/Li interfaces suggest distinct early SEI-forming tendencies. F^−^ species generated from C-F cleavage can coordinate with nearby Li^+^ ions to form LiF-related inorganic precursors, while O^2−^ may contribute to Li_2_O-like products [30,31]. Reduced oxygen-containing fragments, including alkoxide-like species such as OCH_2_CH_3_, OCH_2_CHF_2_, and OCH_2_CF_3_, may further react with Li^+^ to form lithium alkoxides. Other fragments, such as CH_3_CO, CHCO, and unsaturated CH_2_CH/CH_2_CH_2_-type species, may serve as precursors for the organic SEI. Previous studies on carbonate- and fluorinated carbonate-derived SEI formation have shown that reduced organic intermediates, radical species, and alkoxide-type fragments can undergo oligomerization, radical/anionic polymerization, or addition reactions, producing polymeric or semi-carbonate organic SEI components [40,41,42]. On this basis, α-fluorination tends to enhance LiF/Li_2_O-related inorganic precursor formation at the expense of deeper solvent consumption, whereas β-fluorination better preserves molecular integrity and produces a more moderate balance between organic and inorganic precursor formation.

It should also be emphasized that the present models intentionally employ pure solvents and a fixed idealized Li(001) interface in order to isolate the intrinsic reactivity of each fluorinated ester. Therefore, the effects of lithium salts, co-solvents, variations in slab thickness, alternative Li surface structures, and different interfacial molecular configurations were not systematically examined. In practical electrolytes, salts such as LiPF_6_ or LiFSI may alter the Li^+^ solvation structure, solvent orientation, and competition between solvent and anion reduction, thereby changing the accessibility of C-F and C-O bonds and generating additional anion-derived species [43,44]. Differences in slab thickness, surface facets or defects, and molecular adsorption configurations may also affect the absolute charge transfer, reaction-onset times, and minor decomposition pathways. Nevertheless, the same seven-layer Li(001) slab, solvent density, and initial solvent framework were adopted for all six systems to enable a controlled comparison of the α- and β-fluorinated solvents. Moreover, the present 20 ps AIMD trajectories primarily capture the earliest electron-transfer and bond-cleavage events at the initially exposed Li/solvent interface. Therefore, the observed reaction selectivities and fragment distributions should be interpreted as intrinsic early-stage decomposition trends and early SEI precursor characteristics rather than as a complete description of the composition and evolution of a mature multilayer SEI.

## 4. Conclusions

In this work, DFT calculations and AIMD simulations were combined to investigate the intrinsic initial decomposition mechanisms of six pure fluorinated ethyl acetate solvents on the Li(001) surface. By excluding the effects of salts and co-solvents, the simulations isolate how fluorination position and degree regulate frontier-orbital localization, interfacial charge transfer, bond cleavage, and early SEI precursor generation.

The results show that fluorination position plays a decisive role. In α-fluorinated solvents, the electron-accepting region is closely associated with α-C-F/carbonyl-adjacent bonds, leading to preferential C-F cleavage. Increasing α-fluorination from EFA to ETFA increases charge transfer and drives the pathway from single defluorination to cascade C-F, C-O, and C=O cleavage. Thus, α-fluorination efficiently supplies LiF/Li_2_O-related inorganic precursors but can also induce excessive solvent decomposition.

In contrast, β-fluorinated solvents show lower and nonmonotonic reactivity because their LUMO is mainly localized around the carbonyl group and carbonyl-side C-O bond. Their decomposition is therefore initiated primarily by C-O cleavage, while β-C-F cleavage is delayed, configuration-dependent, or absent. TFEA in particular retains intact C-F bonds after initial C-O cleavage, explaining why β-fluorination can maintain reductive stability even in molecules that still contain α-H atoms.

Overall, α- and β-fluorination represent two complementary solvent-design strategies. α-Fluorination favors inorganic interphase precursor formation but risks deep decomposition, whereas β-fluorination better balances molecular stability and limited passivation chemistry. Because this study focuses on pure solvent decomposition on an ideal Li(001) surface within 20 ps AIMD trajectories, the results should be interpreted as intrinsic early-stage solvent-level trends. Future work will extend the simulations to multicomponent electrolyte formulations and more complex Li metal interfaces.

## Figures and Tables

**Table 1:** Summary of representative decomposition pathways and products/intermediates observed for fluorinated ethyl acetate solvents on Li(001) after 20 ps AIMD simulations.

Solvent	Decomposed Molecules	Representative Reaction Step	Cleaved Bonds	Products/Intermediates After 20 ps
EFA	7/17	$CH_{2}\mathrm{FC}(=O)\mathrm{OC}H_{2}CH_{3}$ + 2e^−^ → ${CH_{2}C\left(=O\right)\mathrm{OC}H_{2}CH_{3}}^{-}$ + F^−^	C-F	7 F^−^ + CH_2_COOCH_2_CH_3_-type fragments
EDFA	7/17	$\mathrm{CH}F_{2}C(=O)\mathrm{OC}H_{2}CH_{3}\;+{\;2e}^{-}$ → ${\mathrm{CHFC}(=O)\mathrm{OC}H_{2}CH_{3}}^{-}$ + F^−^	C-F	11 F^−^ + CHCO and OCH_2_CH_3_-type fragments, together with partially/fully defluorinated intermediates
		${\mathrm{CHFC}(=O)\mathrm{OC}H_{2}CH_{3}}^{-}\;+\;e^{-}$ → CHC(=O)OCH_2_CH_3_^−^ + F^−^	C-F	
		${\mathrm{CHC}(=O)\mathrm{OC}H_{2}CH_{3}}^{-}\;+{\;e}^{-}$ → ${\mathrm{CHC}(=O)}^{-}$$\;+\;{\mathrm{OC}H_{2}CH_{3}}^{-}$	carbonyl-side C-O	
ETFA	7/17	$CF_{3}C(=O)\mathrm{OC}H_{2}CH_{3}\;+{\;2e}^{-}$ → ${CF_{2}C(=O)\mathrm{OC}H_{2}CH_{3}}^{-}$ + F^−^	C-F	16 F^−^ + 2 O^2−^ + CC, CCO, and OCH_2_CH_3_-type fragments, together with partially/fully defluorinated intermediates
		${CF_{2}C(=O)\mathrm{OC}H_{2}CH_{3}}^{-}\;+{\;e}^{-}$ → ${\mathrm{CFC}(=O)\mathrm{OC}H_{2}CH_{3}}^{-}$ + F^−^	C-F	
		${\mathrm{CFC}(=O)\mathrm{OC}H_{2}CH_{3}}^{-}\;\;+{\;e}^{-}$ → ${\mathrm{CC}(=O)\mathrm{OC}H_{2}CH_{3}}^{-}$ + F^−^	C-F	
		${\mathrm{CC}(=O)\mathrm{OC}H_{2}CH_{3}}^{-}\;+{\;e}^{-}$ → ${\mathrm{CC}(=O)}^{-}\;+{\;\mathrm{OC}H_{2}CH_{3}}^{-}$	carbonyl-side C-O	
		${\mathrm{CC}(=O)}^{-}\;+{\;e}^{-}$ →$\mathrm{CC}\;+\;O^{2-}$	C=O	
FEA	4/17	$CH_{3}C(=O)\mathrm{OC}H_{2}CH_{2}F\;+\;2e^{-}$ →$CH_{3}C({=O)}^{-}\;+\;\mathrm{OC}H_{2}CH_{2}F^{-}$	carbonyl-side C-O	4 F^−^ + 1 O^2−^ + CH_2_CH_2_, OCH_2_CH_2_, CH_3_CO, and CH_3_COO-type fragments; no persistent F-containing fragments remained
		$\mathrm{OC}H_{2}CH_{2}F^{-}\;+{\;e}^{-}$ → ${\;\mathrm{OC}H_{2}CH_{2}}^{-}\;$+ F^−^	C-F	
		${\mathrm{OC}H_{2}CH_{2}}^{-}\;+{\;e}^{-}$ →$CH_{2}CH_{2}\;+{\;O}^{2-}$	C-O	
DFEA	3/17	$CH_{3}\mathrm{CO}(=O)CH_{2}\mathrm{CH}F_{2}\;+\;2e^{-}$ →$CH_{3}C({=O)}^{-}\;+\;{\mathrm{OC}H_{2}\mathrm{CH}F_{2}}^{-}$	carbonyl-side C-O	2 F^−^ + 1 O^2−^ + CH_3_CO, CH_2_CH, and OCH_2_CHF_2_-type fragments
		${\mathrm{OC}H_{2}\mathrm{CH}F_{2}}^{-}\;+{\;e}^{-}$ → ${\mathrm{OC}H_{2}\mathrm{CHF}}^{-}\;$+ F^−^	C-F	
		${\mathrm{OC}H_{2}\mathrm{CHF}}^{-}\;+{\;e}^{-}$ → ${\mathrm{OC}H_{2}\mathrm{CH}}^{-}\;$+ F^−^	C-F	
		${\mathrm{OC}H_{2}\mathrm{CH}}^{-}\;+{\;e}^{-}$ →$CH_{2}\mathrm{CH}\;+{\;O}^{2-}$	C-O	
TFEA	4/17	${\mathrm{CH}}_{3}\mathrm{CO}(=O)CH_{2}CF_{3}\;+\;2e^{-}$ →$CH_{3}C({=O)}^{-}\;+\;{\mathrm{OC}H_{2}CF_{3}}^{-}$	carbonyl-side C-O	CH_3_CO and OCH_2_CF_3_-type fragments; no F^−^ or O^2−^ detected

## Data Availability

The data presented in this study are available in the article and Supplementary Materials. Additional data are available from the corresponding author upon reasonable request.

## Supplementary Materials

The following supporting information can be downloaded at: https://www.mdpi.com/article/10.3390/nano16130810/s1.
