# Supplementary material for: Fluorination Site and Degree Regulate the Decomposition of Fluorinated Ethyl Acetate Solvents on Lithium Metal: A First-Principles Molecular Dynamics Study
Source: Nanomaterials (Basel). 2026 Jun 30;16(13):810. doi: 10.3390/nano16130810 (PMC13362590; doi:10.3390/nano16130810)
Supplement: Supplementary file 1 [file nanomaterials-16-00810-s001.zip › nanomaterials-4350624-supplementary.pdf]

Supplementary Materials

S1. Detailed construction of pure-solvent/Li-metal interface models

Following the Li metal interface modeling strategy used in our previous studies [30,31], the Li metal substrate was constructed using a seven-layer 4 × 4 Li(001) supercell pre-optimized with VASP. The bottom two Li layers were fixed to mimic bulk metallic lithium. To compare the intrinsic reactivity of different fluorinated ethyl acetate solvents, all interface models were constructed with the same initial solvent framework, average solvent density, and solvent–surface configuration, while only the fluorination position and degree were varied.

A solvent layer containing 17 EDFA molecules was first generated using the Amorphous Cell module in Materials Studio 2020 (BIOVIA Software Inc., San Diego, CA, USA) at a target density of 1.172 g/mL, corresponding to the average density of the six studied solvents (Table S1). Ten amorphous configurations with dimensions of 13.85 Å × 13.85 Å × 15.58 Å were optimized using the Forcite module with the COMPASS III force field, and the lowest-energy configuration was selected as the initial solvent layer. This solvent layer was then placed above the pre-optimized Li(001) surface. A fixed helium buffer layer and an additional vacuum region of approximately 4 Å were introduced along the surface-normal direction to reduce artificial periodic interactions [28], giving a total cell height of approximately 36 Å.

The other five solvent/Li interface models were generated from the EDFA/Li model by changing only the number and position of F atoms and the corresponding H atoms, while keeping the initial solvent skeleton arrangement unchanged. Specifically, the EFA and ETFA models were obtained by decreasing or increasing the number of α-position F atoms, respectively, whereas the FEA, DFEA, and TFEA models were constructed by placing one, two, and three F atoms at the β position, respectively. After atomic substitution, each interface model was further optimized using VASP to remove unphysical short contacts while preserving comparable initial interfacial configurations. The optimized structures were then used as the initial configurations for the AIMD simulations.

Table S1. Experimental densities, average density, and relative deviations of six fluorinated ethyl acetate solvents

Solvent	Experimental Density (298 K, g/mL)	Average Density (g/mL)	Relative Deviation (%)
EFA	1.098	1.172	+6.74
EDFA	1.180	1.172	-0.68
ETFA	1.194	1.172	-1.84
FEA	1.098	1.172	+6.74
DFEA	1.203	1.172	-2.58
TFEA	1.258	1.172	-6.84

Considering that the experimental density of ethyl difluoroacetate (EDFA) (1.180 g/mL) is the closest to the average density of the six fluorinated ethyl acetates (1.172 g/mL) with a relative deviation of only -0.68% (Table S1), the initial structure constructed based on this density is closest to the thermodynamic equilibrium state. Therefore, the Amorphous Cell module of Materials Studio was first used to build an amorphous box containing 17 EDFA molecules at a density of 1.172 g/mL.

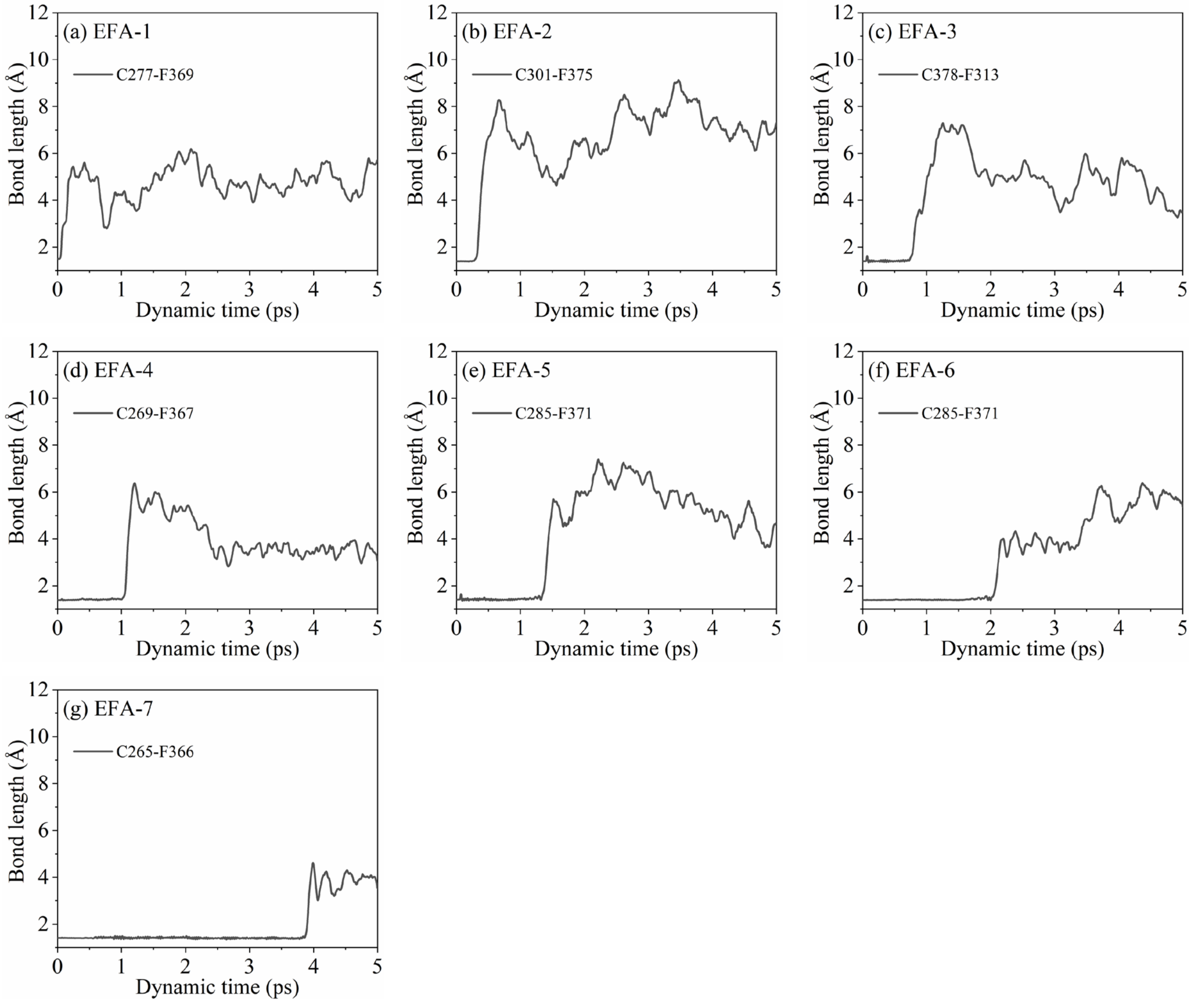

Figure S1. Real-time C-F bond-length evolution of the seven decomposed EFA molecules in the EFA/Li interface system.

All decomposed EFA molecules during the simulation only underwent C-F bond cleavage (a bond length exceeding 2.8 Å was adopted as the criterion for covalent bond cleavage in this study). The maximum bond lengths of other chemical bonds did not exceed 2.0 Å, which is far below the general critical threshold for covalent bond cleavage (2.2-2.5 Å). Therefore, the bond-length evolution curves of other bonds are not shown.

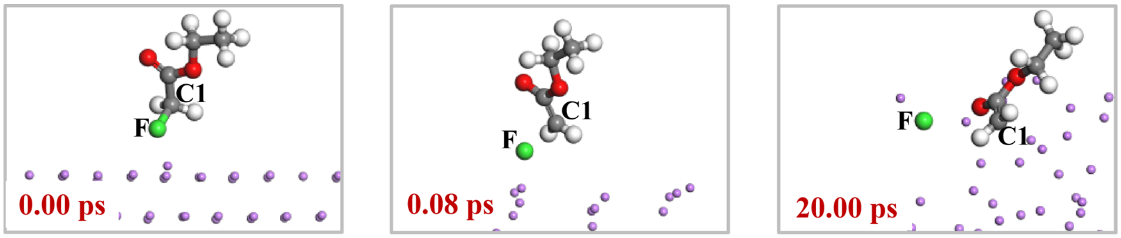

$CH_2FCOOCH_2CH_3$ | C1-F bond breaking | $CH_2COOCH_2CH_3$+F
EFA: -0.44 |e| | EFA: -1.67 |e| | EFA: -1.67 |e|
C1: 0.43 |e|， F: -0.64 |e| | C1: -0.07 |e|, F: -0.89 |e| | C1: -0.15 |e|, F: -0.80 |e|

Figure S2. Snapshots of the decomposition process of the EFA-1 molecule on the lithium metal surface and the corresponding real-time Bader charges. The total charge of the EFA molecule and the Bader charges of the key C1 and F atoms are labeled at selected simulation times.

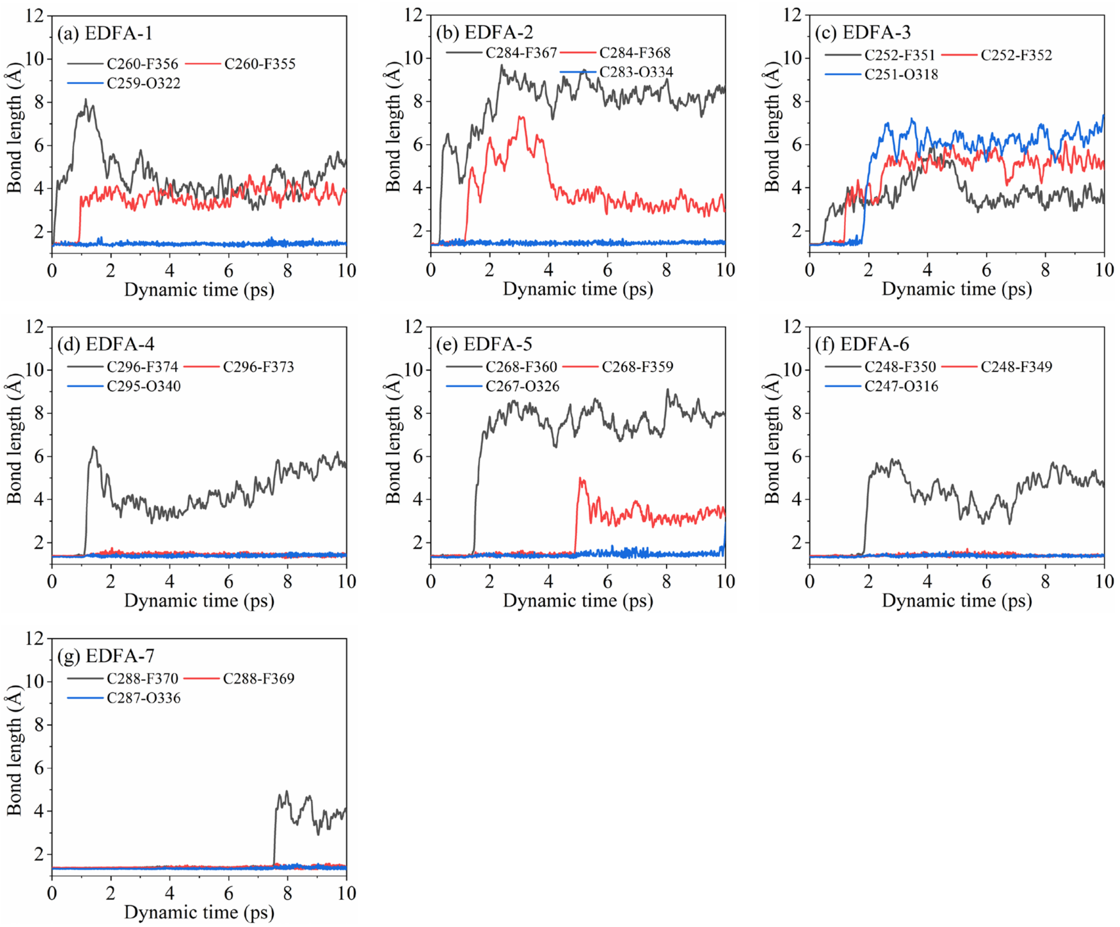

Figure S3. Real-time key bond-length evolution of the seven decomposed EDFA molecules in the EDFA/Li interface system. The black and red curves represent the two C-F bonds of the EDFA molecule, respectively, and the blue curve represents the carbonyl-side C-O bond.

EDFA-1, EDFA-2, and EDFA-5 molecules underwent cleavage of both C-F bonds; EDFA-4, EDFA-6, and EDFA-7 molecules only underwent cleavage of one C-F bond; and the EDFA-3 molecule exhibited the most complete decomposition, with cleavage of both C-F bonds and the carbonyl-side C-O bond. Therefore, the EDFA-3 molecule was selected as an example to demonstrate the complete decomposition pathway of EDFA on the lithium metal surface (**Figure S4**).

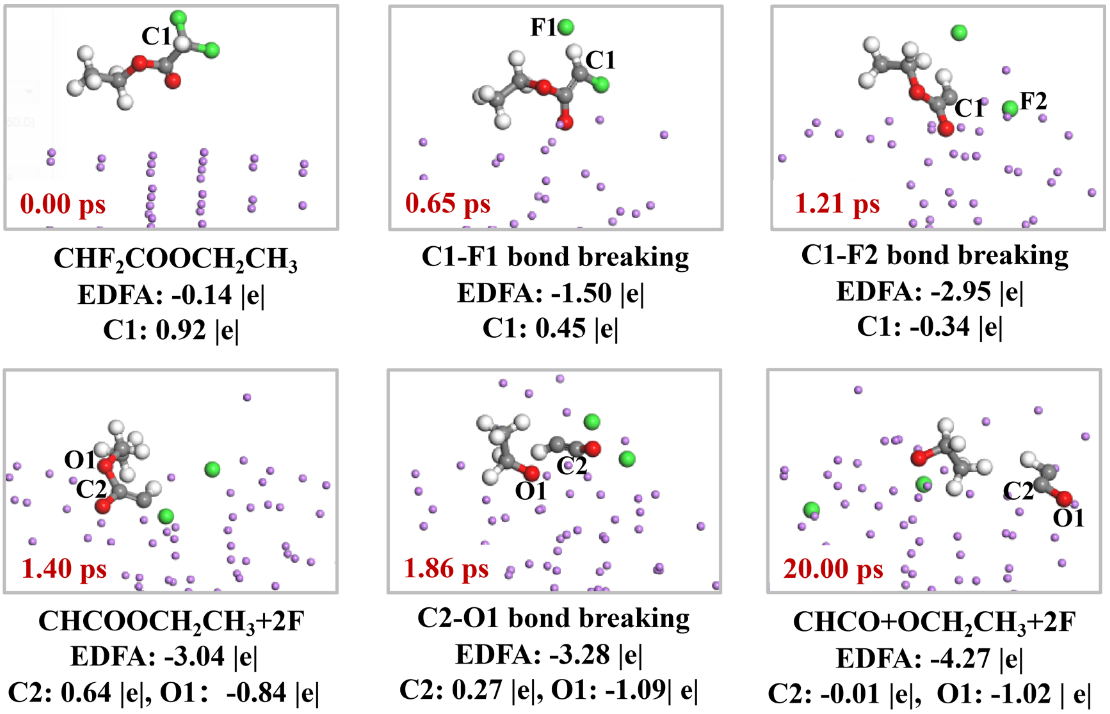

Figure S4. Snapshots of the decomposition process of the EDFA-3 molecule on the lithium metal surface and the corresponding real-time Bader charges. The total charge of the EDFA molecule and the Bader charges of the key C1, C2 and O1 atoms are labeled at selected simulation times.

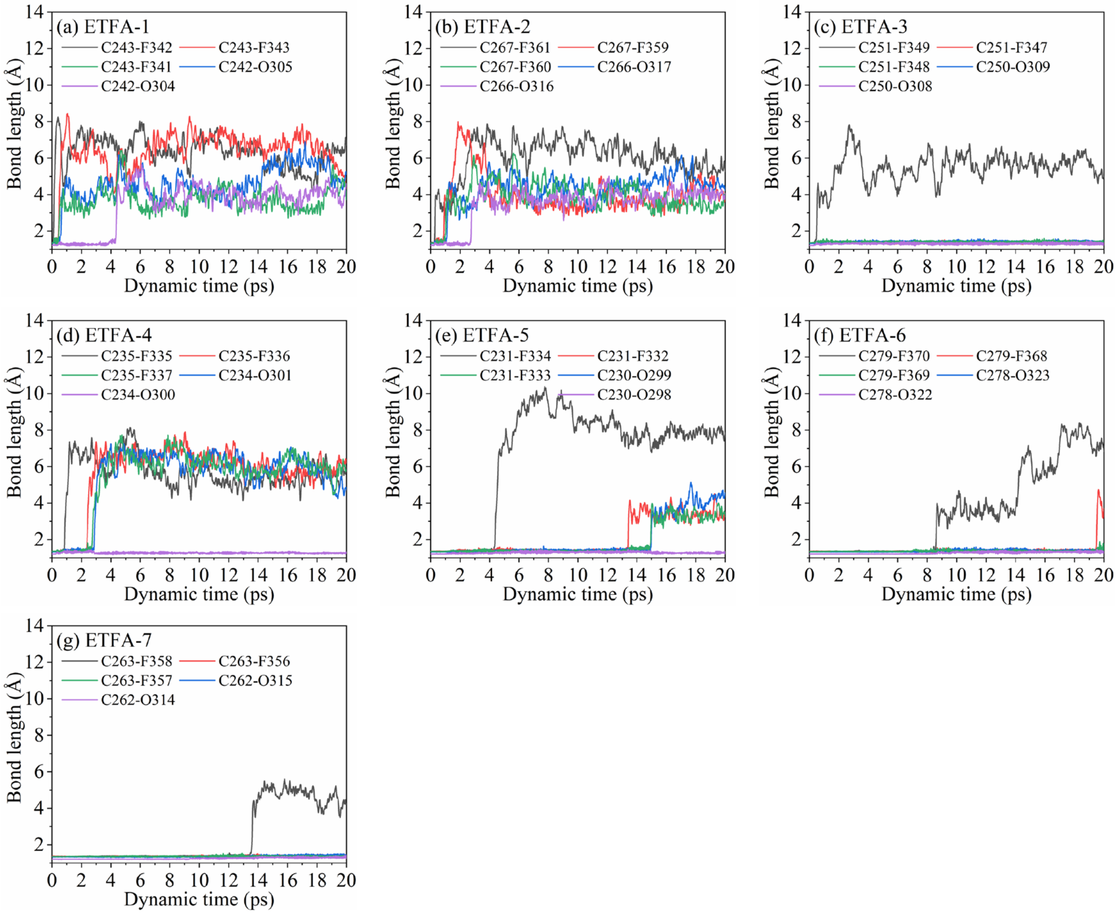

Figure S5. Real-time key bond-length evolution of the seven decomposed ETFA molecules in the ETFA/Li interface system. The black, red, and green curves represent the three C-F bonds of the ETFA molecule, respectively; the blue curve represents the carbonyl-side C-O bond; and the purple curve represents the C=O double bond.

ETFA-1 and ETFA-2 molecules underwent complete decomposition, while the other molecules only underwent partial decomposition. Therefore, the ETFA-1 molecule was selected as an example to demonstrate the complete decomposition pathway of ETFA on the lithium metal surface (**Figure S6**).

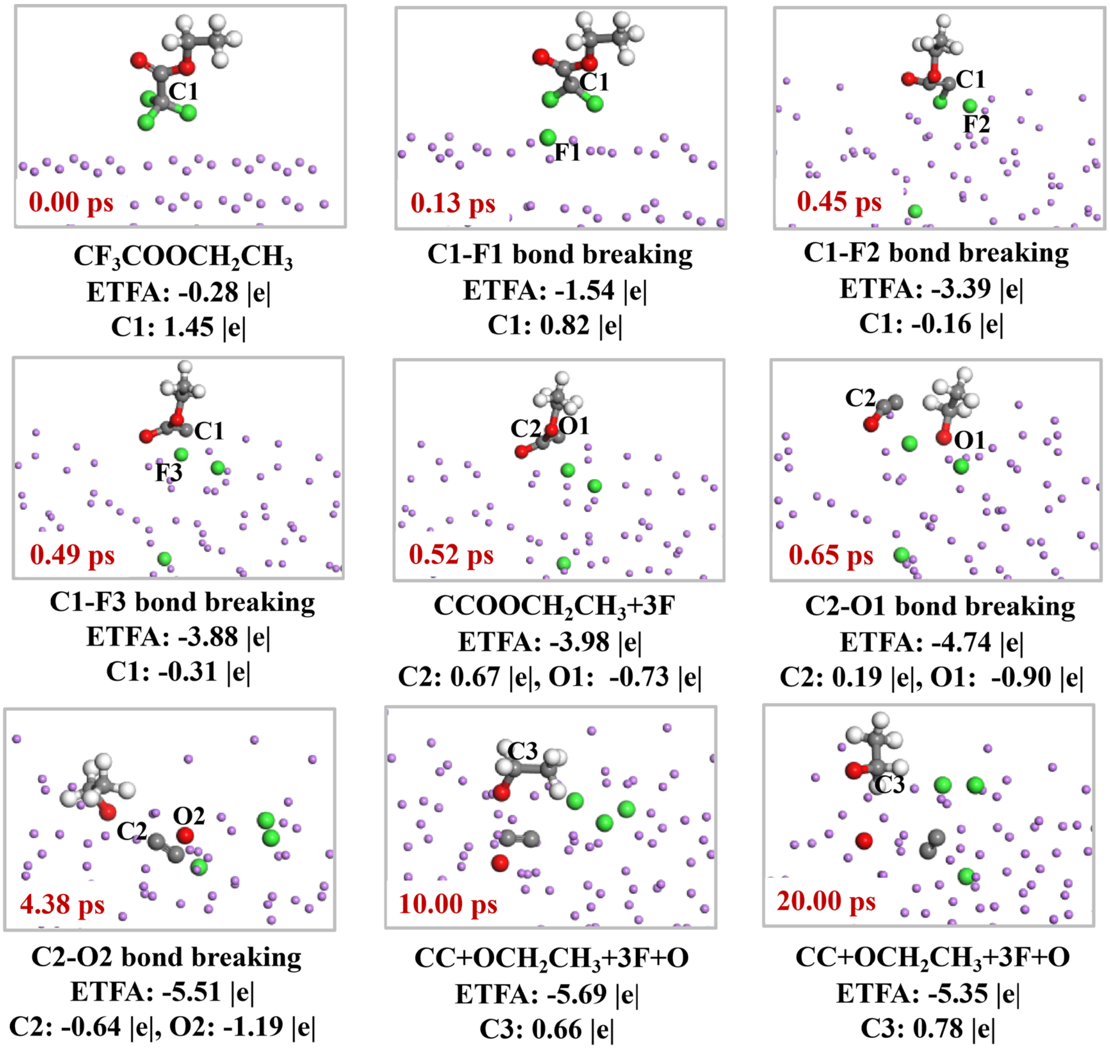

Figure S6. Snapshots of the decomposition process of the ETFA-1 molecule on the lithium metal surface and the corresponding real-time Bader charges. The total charge of the ETFA molecule and the Bader charges of the key C1, C2, C3, O1 and O2 atoms are labeled at selected simulation times.

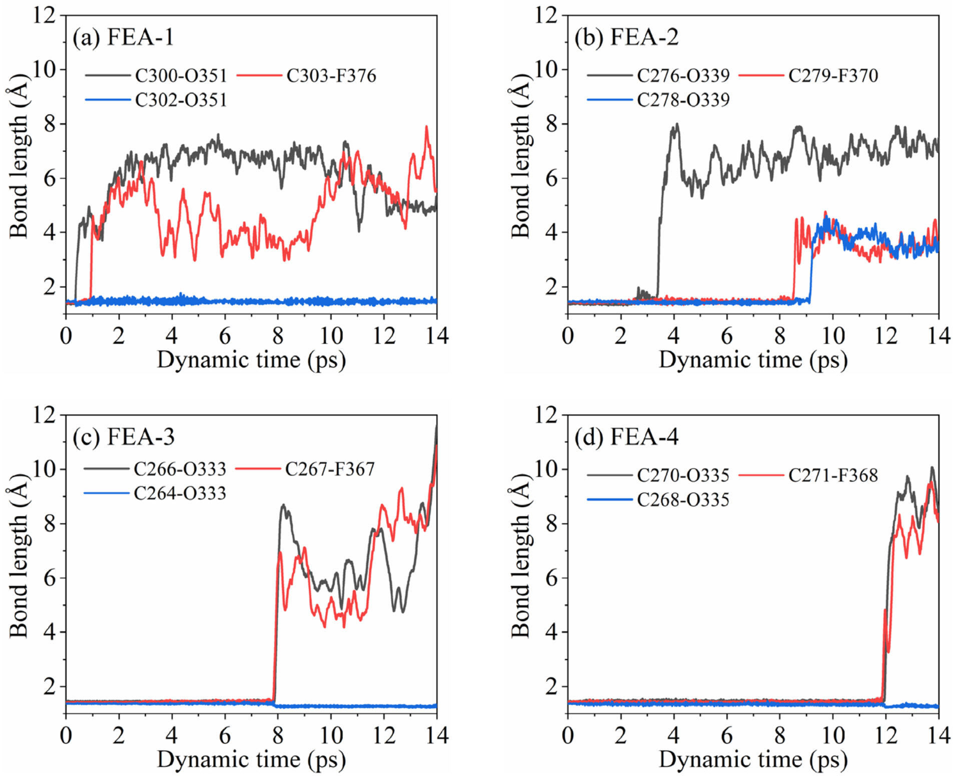

Figure S7. Real-time key bond-length evolution of the four decomposed FEA molecules in the FEA/Li interface system. The black, red, and blue curves represent the carbonyl-side C-O bond, C-F bond, and ethoxy-side C-O bond of the FEA molecule, respectively.

Among the four decomposed FEA molecules, two bond-cleavage sequences were observed. FEA-1 and FEA-2 preferentially underwent carbonyl-side C-O bond cleavage followed by C-F bond cleavage (Figures S8 and S9). In particular, FEA-2 exhibited the most extensive decomposition, with additional cleavage of the ethoxy-side C-O bond after the initial C-O and C-F bond-breaking events. Therefore, FEA-2 was selected as the representative molecule to illustrate the C-O-first decomposition pathway of FEA in the main text.

In contrast, FEA-3 and FEA-4 showed an alternative cleavage sequence, in which C-F bond cleavage occurred before rapid C-O bond cleavage. These C-F-first events appeared at later simulation times than the initial C-O cleavage observed for FEA-1 and FEA-2. The detailed snapshots of FEA-3 shown in Figure S10 indicate that this pathway is associated with a specific adsorption configuration in which the fluorinated ethoxy group faces the Li metal surface. Therefore, the FEA decomposition behavior is mainly characterized by an earlier-onset C-O-first pathway, whereas the C-F-first sequence can be regarded as an adsorption-configuration-dependent alternative pathway.

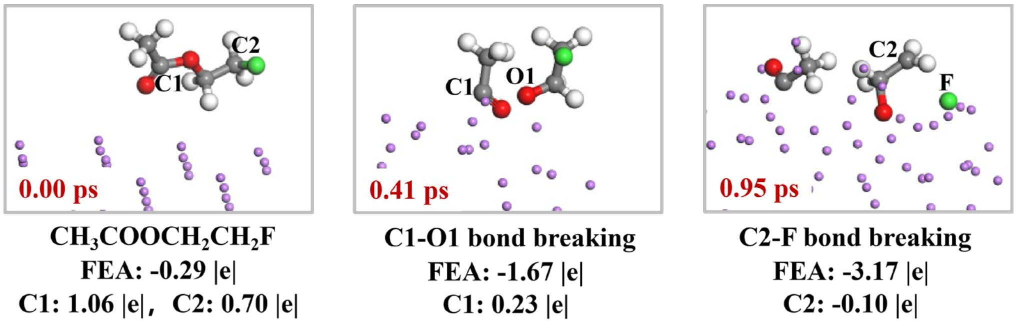

Figure S8. Snapshots of the decomposition process of the FEA-1 molecule on the lithium metal surface and real-time Bader charges. The total charge of the FEA molecule and the Bader charge values of the key C atoms at selected moments are labeled below the corresponding snapshots.

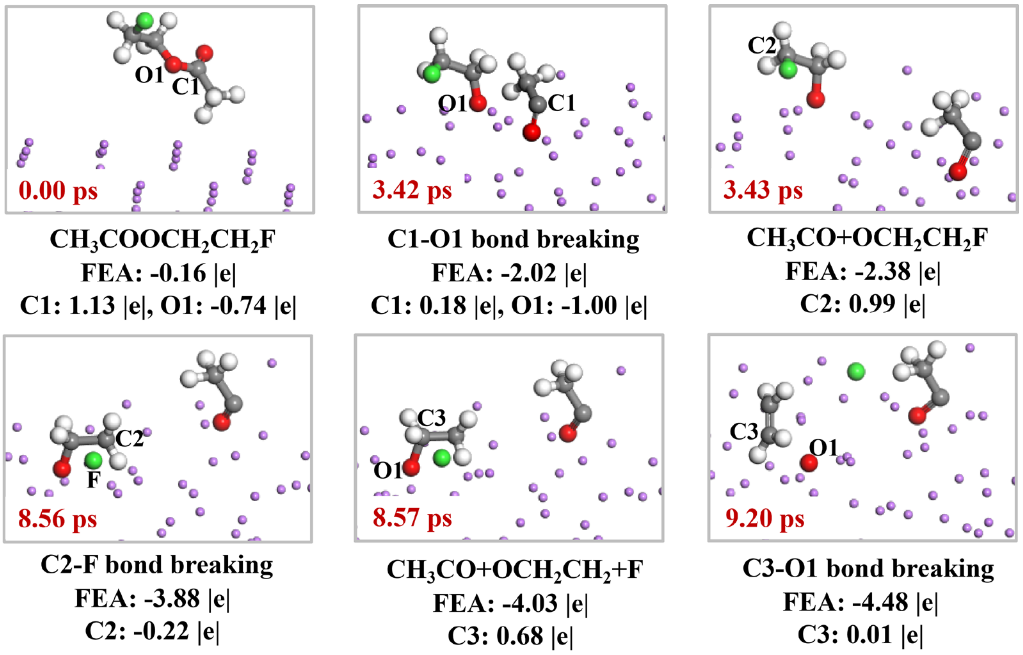

Figure S9. Snapshots of the decomposition process of the FEA-2 molecule on the lithium metal surface and the corresponding real-time Bader charges.

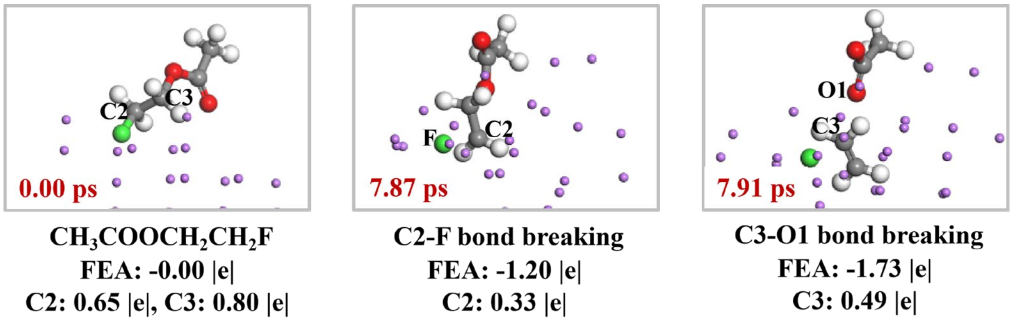

Figure S10. Snapshots of the decomposition process of the FEA-3 molecule on the lithium metal surface and the corresponding real-time Bader charges.

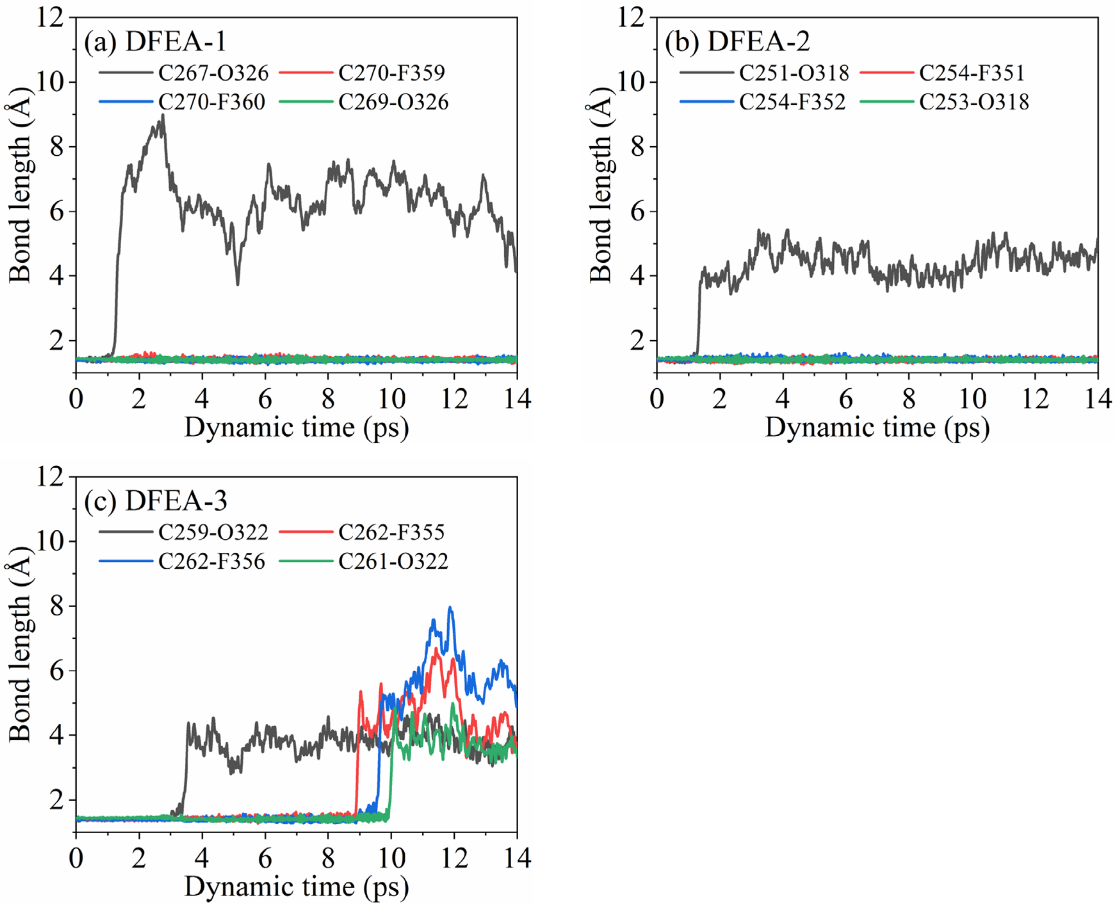

Figure S11. Real-time key bond-length evolution of the three decomposed DFEA molecules in the DFEA/Li interface system. The black curve represents the carbonyl-side C-O bond of the DFEA molecule, the red and blue curves represent the two C-F bonds, respectively, and the green curve represents the ethoxy-side C-O bond.

DFEA-1 and DFEA-2 molecules only underwent carbonyl-side C-O bond cleavage; the DFEA-3 molecule exhibited the most complete decomposition, with cleavage of both C-O bonds and both C-F bonds. Therefore, the DFEA-3 molecule was selected as an example to focus on the complete decomposition pathway of DFEA on the lithium metal surface (**Figure S12**).

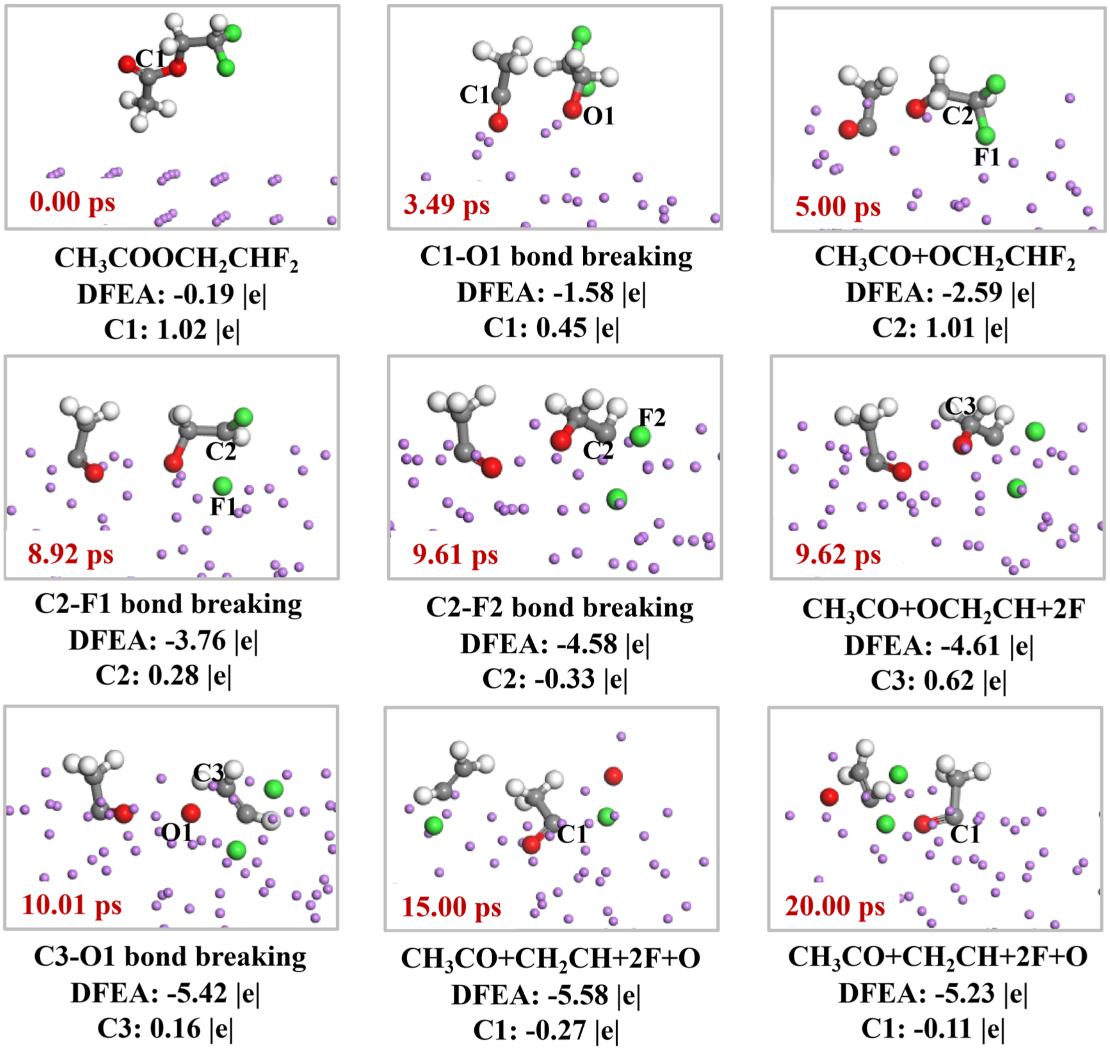

Figure S12. Snapshots of the decomposition process of the DFEA-3 molecule on the Li metal surface and the corresponding real-time Bader charges. The total charge of the DFEA molecule and the Bader charge values of the key C atoms at selected moments are labeled below the corresponding snapshots.

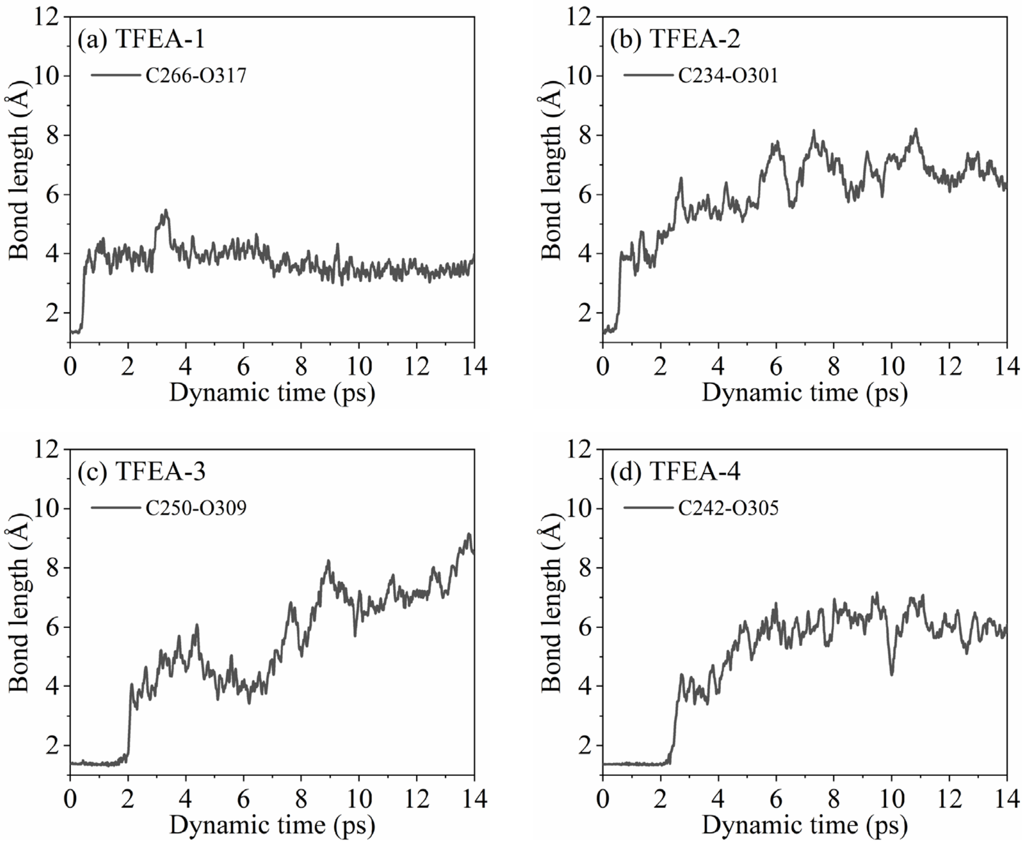

Figure S13. Real-time C-O bond-length evolution of the four decomposed TFEA molecules in the TFEA/Li interface system.

All four TFEA molecules only underwent carbonyl-side C-O bond cleavage, exhibiting the lowest decomposition degree among the three β-fluorinated ethyl acetates (FEA, DFEA, TFEA) and a significantly lower decomposition depth than that of the α-fluorinated ETFA molecule.

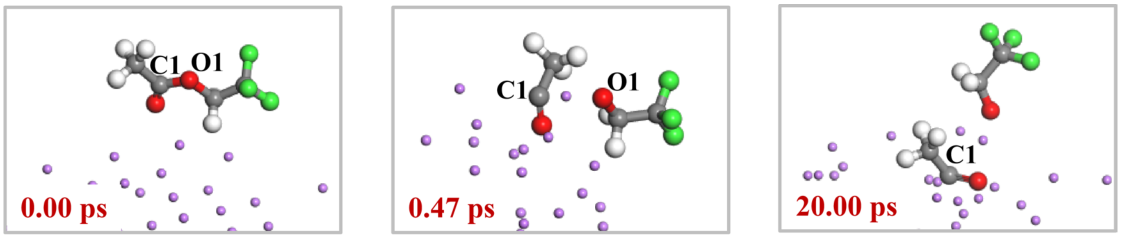

$CH_3COOCH_2CF_3$
TFEA: -0.28 |e|
C1: 1.13 |e|

C1-O1 bond breaking
TFEA: -1.58 |e|
C1: 0.45 |e|

CH_3CO+OCH_2CF_3
TEFA: -2.53 |e|
C1: -0.31 |e|

Figure S14. Snapshots of the decomposition process of the TFEA-1 molecule on the Li metal surface and the corresponding real-time Bader charges. The total charge of the TFEA molecule and the Bader charge values of the key C atoms at selected moments are labeled below the corresponding snapshots.
